# 

**Published:** 1845-07

**Authors:** 


### CONTENTS

OF THE

### MEDICAL EXAMINER.

NEW SERIES—NO. VII.—JULY, 1845.

### ORIGINAL COMMUNICATIONS.

Contributions to General Surgery. By W. H. Elliot, D. D. S., Fellow of the American Society of Dental Surgeons. Plattsburgh, N. Y. - - - - - - - - - - - - - 389

Experiments on the hearts of the Sturgeon, Snapping Turtle, and Frog, when removed from the body. By F. G. Smith, jr., M. D., Lecturer on Physiology in the Philadelphia Medical Association, &c. (Communicated by Professor Dunglison,) - - 393

### CLINICAL LECTURES AND REPORTS.

PHILADELPHIA HOSPITAL,

*Saturday, February* 1, 1845.

Clinic of Professor Dunglison.

Paraplegia, - - - - - - - - - - - - - - - 395

Chorea, - - - - - - - - - - - - - - - 396

Salaam convulsion, - - - - - - - - - - - - 399

Discoloration from Nitrate of Silver, - - - - - - - 400

Pathological specimens.—Open Foramen Ovale, and Pulmonary Consumption, in a colored female ninety-eight years of age, - 401

Ulceration of the Rectum, - - - - - - - - - - 403

### BIBLIOGRAPHICAL NOTICES.

An Apology for the Nerves; or their influence and importance in health and disease. By Sir George Lefevre, M. D., Fellow of the Royal College of Physicians, late Physician to the British Embassy at the Court of St. Petersburg, &c. &c. - - - - 404

Principles and Illustrations of Pathological Anatomy; being a complete series of Colored Lithographic Drawings. By J. Hope, M. D., F. R. S., Physician to the St. Mary-Le-Bone Infirmary; Mem. Hon. De La Société De Statistique Universelle; Extraord. Mem. and formerly Pres. of the Royal Med. Soc. Ed., etc. First American Edition. Edited by L. M. Lawson, M. D., Professor of General and Pathological Anatomy and Physiology in Transylvania University, - - - - - - - - - 411

Transactions of the New York State Medical Society, Albany, 1845, - 413

Manual of Orthopedic Surgery, being a Dissertation which obtained the Boylston prize for 1844, on the following question: "In what cases and to what extent is the division of Muscles, Tendons, or other parts proper for the relief of deformity or lameness?" By Jacob Bigelow, M. D., 8vo., pages 211, with several plates, - 415

The Dispensatory of the United States of America. By George B. Wood, M. D., Professor of Materia Medica and Pharmacy in the University of Pennsylvania; one of the Physicians of the Pennsylvania Hospital, &c. &c.; and Franklin Bache, M. D., Professor of Chemistry in Jefferson Medical College of Philadelphia; one of the Vice Presidents of the American Philosophical Society, &c. &c. Sixth Edition, carefully Revised, - 416

Copland's Dictionary of Practical Medicine, - - - - - - 416

### EDITORIAL.

New Medical Journals, - - - - - - - - - - 417
The Missouri Medical and Surgical Journal, - - - - - - 418
The Buffalo Medical Journal, - - - - - - - - 418
National Convention of Physicians, - - - - - - - - 419
Transylvania University and the Navy, - - - - - - 419

### RECORD OF MEDICAL SCIENCE.

Case of Lithotomy in the Female, with remarks; reported by A. Baker, Jr., M. D., of Norwich, Chenango County, N. Y. - - 420
Case of Hydrophobia, - - - - - - - - - - - 423
Notices of the Lunatic Asylums of Paris, &c. In a letter addressed to John Forbes, M. D. F. R. S. By John Conolly, M. D. Fellow of the Royal College of Physicians, Physician to the County Lunatic Asylum at Hanwell, - - - - - - - - 426
Birth of Four Children, - - - - - - - - - - 435
The History of a Human Anencephalus, - - - - - - - 435
On the Convulsions of New-born Children, - - - - - - 435
Foreign Body in the Rectum, - - - - - - - - - 436
Sir William Burnett's Patent, - - - - - - - - - 437
Case of Purpura Hæmorrhagica, - - - - - - - - - 438
The treatment of Syphilitic Affections, - - - - - - - 439
New application of Electricity to Surgery, - - - - - - - - 441
Professor Corneliani on the Proximate Cause and Treatment of Chlorosis, - - - - - - - - - - - - - - - 443
M. Cazenave on the Different Sorts of Caustics, - - - - 443
Fluid caustics, - - - - - - - - - - - - 444
Ethnological Society, April 23, - - - - - - - - - 445
Case of remarkable hypertrophy of the fingers in a girl; with a notice of some similar cases, - - - - - - - - - 447
Description of a malformation of the duodenum. By Robert Boyd, M. D. - - - - - - - - - - - - - 448

A case of abscess in the groin, attended with symptoms of Hernia, from which two lumbrici teretes and afterwards fæcal matter were discharged, and the patient recovered. By Thomas Howell, Esq., of Risborough, - - - - - - - - - - 448
On Simple Ulceration of the Os Uteri, - - - - - - 449
On the Effects of Blood as an Antidote to Arsenic, - - - - 453
Poisoning by Cherry Kernels, - - - - - - - - . 425

